# A Review of Phytoplankton Sinking Rates: Mechanisms, Methodologies, and Biogeochemical Implications

**DOI:** 10.3390/biology15020130

**Published:** 2026-01-12

**Authors:** Jie Zhu, Jiahong Cheng, Jiangning Zeng, Wei Zhang, Chenggang Liu, Kokoette Sunday Effiong, Qiang Hao

**Affiliations:** 1 Ocean College, Zhejiang University, Zhoushan 316000, China; zhujiezd@zju.edu.cn; 2 Second Institute of Oceanography, Ministry of Natural Resources, Hangzhou 310012, China; 3 Key Laboratory of Marine Ecosystem Dynamics, Ministry of Natural Resources, Hangzhou 310012, China; 4 Zhejiang Key Laboratory of Nearshore Engineering Environment and Ecological Security, Hangzhou 310012, China; 5 Marine Biology Department, Faculty of Biological Sciences, Akwa Ibom State University, Uyo P.O. Box 1167, Nigeria

**Keywords:** phytoplankton, sinking rates, aggregation, biological carbon pump, SETCOL

## Abstract

Phytoplankton sinking is a pivotal process within the biological carbon pump that drives the vertical transport of organic carbon in the ocean. Its rates and underlying mechanisms directly influence the efficiency of the global carbon cycle and the potential for long-term sequestration. This review synthesizes current knowledge of phytoplankton sinking, encompassing buoyancy regulation mechanisms, environmental and physiological controls, methodological approaches such as settling column (SETCOL), and comparative evidence from laboratory and field studies. The aim is to elucidate the regulatory processes governing sinking and to provide a foundation for improving ecological models and refining estimates of carbon export. Evidence demonstrates that sinking rates vary considerably among phytoplankton groups, with nutrient limitation and aggregation emerging as critical modulators of export efficiency. By integrating results from experimental and in situ research, this review identifies unresolved questions and highlights priority areas: (1) quantitative coupling between aggregation and carbon flux; (2) mechanistic understanding of group-specific sinking responses; (3) integration of novel technologies, including in situ imaging and high-resolution modeling with established methods; and (4) development of interdisciplinary frameworks. Overall, this review consolidates current knowledge and underscores phytoplankton sinking as a crucial yet insufficiently resolved process within the marine carbon cycle.

## Simple Summary

Phytoplankton are microscopic algae that form the base of marine food webs. They do not simply float in the ocean; many sink, and their sinking speed changes with their shape, size, health, and environmental conditions such as light, temperature, and nutrient availability. Additionally, the biomass and community composition of phytoplankton are also critical factors influencing sinking speed, as different species and functional groups may exhibit different sinking rates. This review summarizes how phytoplankton adjust their buoyancy by regulating salts and water within vacuoles, changing the proportions of lipids and carbohydrates, forming chains or colonies, and producing sticky substances that promote aggregation into marine snow. It also compares common methods used to measure sinking rates, from basic settling columns to modern optical and imaging techniques, highlighting the value of combining different approaches for more accurate results. Laboratory and field studies show that nutrient stress and late bloom stages can cause either an increase in the number of sinking particles or aggregates, or an increase in the sinking velocity of individual particles, thereby enhancing the vertical transport of organic matter to deeper waters and influencing the efficiency of carbon sequestration. Because this downward flux drives the ocean’s biological carbon pump, which helps store carbon in the deep ocean, understanding how and why phytoplankton sink is essential for improving climate models, managing harmful algal blooms, and protecting marine ecosystems.

## 1. Introduction

Phytoplankton are the primary producers in marine ecosystems, accounting for approximately half of global primary productivity and playing a pivotal role in the global carbon cycle and climate regulation [1,2,3]. Through photosynthesis, they fix atmospheric CO_2_ into organic carbon, thereby driving the operation of the biological carbon pump (BCP), which governs ocean carbon sequestration and its redistribution across the Earth system [4,5]. It is estimated that phytoplankton fix about 5 gigatons of carbon annually, of which approximately 10–20% sinks below the euphotic zone in the form of particulate organic carbon (POC) [3,6,7]. However, only around 1% of the exported POC ultimately reaches the seafloor and becomes permanently buried in sediments [2,8,9]. The efficiency of this process is modulated by a suite of interacting factors, including phytoplankton community composition, cell size, sinking velocity, and the interplay between physical and biological processes in the ocean [10,11,12]. The complexity of these mechanisms presents a significant challenge to accurately quantifying carbon export efficiency and predicting ocean carbon sequestration potential, rendering this topic a central and contested issue in contemporary marine ecology and biogeochemistry.

Phytoplankton sinking rates play a critical role in regulating the efficiency of the ocean biological pump, yet these rates vary considerably across taxonomic groups and physiological states [13]. Sinking velocity should not be considered in isolation but must be interpreted in the context of species identity and functional traits. Generally, non-motile taxa with dense mineral ballasts exhibit higher sinking velocities [14,15]. For instance, diatoms, characterized by heavy siliceous frustules, typically sink faster than other microalgae, with large species such as *Coscinodiscus* exhibiting rates exceeding 10 m d^−1^ under specific conditions [16,17,18]. In contrast, smaller or motile phytoplankton, such as dinoflagellates and flagellates, often exhibit much lower sinking velocities (typically <1 m d^−1^) or maintain position in the water column through active swimming [19]. Furthermore, cyanobacteria (e.g., *Microcystis*, *Trichodesmium*) possess gas vesicles that provide positive buoyancy, allowing them to float or regulate their vertical position independent of Stokesian settling [20]. However, these taxon-specific baselines are dynamically modulated by physiological mechanisms. Phytoplankton can actively adjust their buoyancy by regulating intracellular ion concentrations, synthesizing low-density lipids, or altering carbohydrate ballasting in response to environmental drivers like light and nutrient availability [16]. Consequently, sinking is a plastic trait; for example, nutrient-stressed cells may accelerate their descent to access deep-water resources or form aggregates, significantly enhancing vertical flux beyond individual cell velocities.

However, due to the adhesive properties of their cell surfaces, they can aggregate into larger particles via flocculation or become repackaged into fast-sinking fecal pellets through zooplankton grazing, significantly enhancing their sinking rates and accelerating the transfer of carbon to the deep ocean [12,21,22,23]. On the other hand, many phytoplankton possess physiological mechanisms to regulate their own buoyancy. By adjusting intracellular ion concentrations or synthesizing low-density compounds, they can actively regulate their density and control sinking behavior under varying environmental conditions [24,25,26]. For example, large diatoms or species with dense cell walls can sink at rates of several tens of meters per day under laboratory or controlled conditions, efficiently exporting POC to deeper layers [7,27,28]. However, such high sinking velocities are typically transient or species-specific and are rarely sustained at the population or bloom scale in natural marine environments, where physical mixing, grazing, and physiological regulation act to moderate net sinking rates. In contrast, smaller phytoplankton such as dinoflagellates and cyanobacteria tend to sink more slowly and are more likely to be remineralized in surface waters, thereby contributing less to long-term carbon export [29]. Despite extensive research, the relative contributions of flocculation, fecal pellet production, and physiological buoyancy regulation to vertical carbon flux remain debated. Therefore, elucidating the multiple mechanisms underlying phytoplankton sinking is essential for advancing our understanding of the biological carbon pump and improving the predictive accuracy of ocean carbon sequestration potential.

Recent studies have increasingly revealed the highly variable nature of phytoplankton sinking rates, showing that short-term environmental fluctuations can lead to marked changes in sinking behavior, thereby influencing the efficiency of the biological carbon pump [18,26,30]. Evidence suggests that under nutrient-limited conditions or during the post-bloom phase, phytoplankton can rapidly form mucilaginous aggregates, resulting in abrupt increases in particle sinking rates [31,32,33]. Similarly, certain large phytoplankton such as diatoms tend to increase cell density or produce resting spores during late growth stages, thereby accelerating their descent to escape unfavorable conditions [13,21]. These enhanced sinking processes not only expedite vertical carbon export but also suppress the remineralization of organic carbon in surface waters, thereby increasing the efficiency of long-term carbon sequestration. Collectively, these findings demonstrate that phytoplankton sinking rates are not fixed but dynamically regulated by physiological status and environmental drivers. Understanding and quantifying these dynamic processes is now a critical frontier in marine carbon cycle research.

This review systematically evaluates recent advances in phytoplankton sinking research, with emphasis on three key aspects: (1) a synthesis of sinking mechanisms and conceptual frameworks, including classical Stokes’ law and physiological buoyancy regulation; (2) a critical assessment of methodological approaches for measuring sinking rates, encompassing both laboratory- and field-based techniques, with comparative analysis of their advantages, limitations, and applications; and (3) a comparative evaluation of phytoplankton sinking behaviors under varying environmental conditions, integrating evidence from field observations and laboratory experiments to explore how factors such as light availability and nutrient status influence sinking dynamics, which in turn affect the efficiency of the biological carbon pump and the potential for long-term carbon sequestration. By synthesizing progress across these domains, this review identifies the key factors and dynamic processes that govern phytoplankton sinking rates and provides a scientific foundation for advancing our understanding of the biological carbon pump. Ultimately, it offers theoretical support for improving projections of ocean carbon sink functions under future climate scenarios.

## 2. Principles of Phytoplankton Sinking

Rapid sinking of phytoplankton is a key pathway for efficient carbon sequestration. In contrast, slow sinking often leads to phytoplankton cells being degraded by bacteria or consumed by zooplankton, causing organic carbon to remain in the upper ocean and be recycled, thereby limiting its contribution to deep-sea carbon storage [30,34,35,36]. Therefore, a comprehensive understanding of the mechanisms governing phytoplankton sinking is essential for assessing the ocean’s carbon sequestration potential.

In 1851, Stokes proposed the classical Stokes’ law to describe the sinking behavior of particles in a fluid:

*v* = 2*gr*^2^ (*ρ_c_* − *ρ_w_*)(9*η*)^−1^, (1)

where *v* is the sinking velocity, *g* is the gravitational acceleration, *r* is the radius of the spherical particle, *ρ_c_* and *ρ_w_* are the densities of the particle and the surrounding fluid, respectively, and *η* is the dynamic viscosity of the fluid. The number 9 in the denominator is a dimensionless constant derived from the analytical solution of the Navier-Stokes equations under low Reynolds number (Stokes flow) conditions. According to this equation, the sinking velocity (*v*) is directly proportional to the square of the particle radius (*r*^2^) and the density difference (*ρ_c_* − *ρ_w_*), and inversely proportional to the fluid viscosity (*η*). Stokes’ law provides a foundational theoretical framework for studying the sedimentation of particles in aquatic systems and has been widely applied to estimate the sinking behavior of phytoplankton cells, mineral particles, and organic aggregates. It remains a vital tool for understanding the ocean carbon cycle and evaluating the efficiency of the biological pump. However, Stokes’ law assumes that particles are smooth, rigid spheres and applies under low Reynolds number conditions. Under such conditions, viscous forces dominate over inertial forces, meaning that phytoplankton sinking occurs slowly and steadily, without turbulence or wake formation. These assumptions introduce limitations when predicting the sinking velocities of marine particles, which often have irregular shapes, heterogeneous compositions, and occur under more complex hydrodynamic regimes.

Most phytoplankton and their associated particles in the ocean exhibit substantial structural complexity. For example, chain-forming diatoms such as *Chaetoceros* and flat discoid diatoms such as *Coscinodiscus* are typically non-spherical, loosely structured, and often possess porous surfaces or mucilaginous coatings. These characteristics result in sinking velocities that are significantly lower than those of equivalent-volume spheres [24]. Moreover, extracellular substances such as transparent exopolymer particles (TEPs) can form adhesive layers or surface appendages, markedly increasing the hydrodynamic drag and further reducing the effective sinking velocity of particles [37]. As a result, the sinking behavior of natural marine particles often deviates substantially from the idealized conditions assumed by Stokes’ law [35,38].

To address the limitations, subsequent theoretical work has proposed various extensions and modifications. Oseen [39] was the first to incorporate fluid inertia, extending the applicability of Stokes’ law to intermediate Reynolds numbers. Brenner [40] further accounted for the effect of non-spherical particle geometry on the drag coefficient by introducing a ‘shape factor’ to correct the model. At the structural level, Mandelbrot [41] introduced the use of fractal dimension to describe the porous architecture of particle aggregates, providing a mathematical foundation for modeling aggregate sinking. Building on this, Logan and Wilkinson [42] developed a drag-density coupling model based on fractal geometry, incorporating the effects of porosity and surface complexity on hydrodynamic resistance.

In the context of diatom sinking, Miklasz and Denny [38] proposed a modified Stokes model that separately modeled the densities of the frustule and cytoplasm, and introduced a parameter to account for the non-uniform thickness of the silica shell-more accurately representing diatom morphology. More recently, Laurenceau-Cornec et al. [43] proposed a porosity-drag coupling model that systematically integrates internal porosity, surface features, and ballast components (e.g., calcium carbonate or silicate minerals) to estimate the combined effect on particle sinking velocity. Experimental evidence indicates that aggregates containing inorganic ballast such as calcified coccolithophores, exhibit significantly higher sinking velocities due to increased structural density and lower porosity, compared to organic-dominated aggregates.

In summary, Stokes’ law provides the fundamental framework for understanding particle sinking, but its direct application to natural phytoplankton particles is limited by their complex morphology and multiscale structures. Extending the law through fractal modeling, shape correction, and the incorporation of ecological regulation mechanisms is necessary to improve accuracy. These refinements enhance the reliability of sinking velocity estimations and establish theoretical and methodological foundations for predicting ocean carbon flux, developing particle flux models, and evaluating feedbacks in the global carbon cycle.

## 3. Mechanisms of Phytoplankton Sinking

The vertical sinking behavior of phytoplankton is highly variable across species and environmental conditions [30,44,45,46]. Phytoplankton sinking is widely recognized to be closely linked to physiological state, primarily through adjustments in cell density, morphology, and intracellular composition. Currently, buoyancy regulation mechanisms are generally understood to be based on changes in cell density, involving processes such as selective ion uptake [47], production of organic osmolytes [48], vacuole regulation [25], ballasting effects of accumulated carbohydrates [49], lipid storage [50], and periodic changes in cell volume [51]. In addition, environmental factors such as temperature, salinity [16], irradiance [52], reproductive status, and the history of nutrient exposure [53] have been shown to influence phytoplankton sinking rates. The following section summarizes the most commonly recognized physiological mechanisms through which phytoplankton regulate their buoyancy.

### 3.1. Selective Ion Uptake

Selective ion uptake represents a fundamental physiological mechanism by which phytoplankton regulate cell density and buoyancy. Early studies demonstrated that the centric diatom *Ditylum brightwellii* lowers its density by replacing heavier cations (Ca^2+^, Mg^2+^) with lighter ones (Na^+^, K^+^), thereby enhancing flotation [47,54]. In the large dinoflagellate *Pyrocystis noctiluca*, vacuoles occupying up to ~95% of the cell volume contain dilute solutions with markedly low concentrations of SO_4_^2−^, Ca^2+^, and Mg^2+^, compensated largely by NaCl, thereby lowering cell sap density by 2.0–3.1 mg mL^−1^ relative to seawater. Additional NH_4_^+^ accumulation further decreases density by ~0.2 mg mL^−1^, producing positive buoyancy, while loss of metabolic activity leads to rapid sinking at 30–40 cm h^−1^ [55,56]. Recent studies highlight the highly dynamic nature of this regulation. Nutrient repletion can reverse diatom sinking within hours through rapid physiological responses, including the reactivation of ion pumps (e.g., H^+^-ATPase), net uptake of lighter ions (K^+^, Na^+^), and associated vacuolar expansion, all of which reduce average cellular density [18,49]. At the same time, the metabolic shift from nutrient-limited conditions, which are often characterized by the accumulation of dense carbohydrate reserves, toward active growth alleviates intracellular carbon ballasting and further promotes neutral or positive buoyancy [49,57]. Together, these ion-mediated and metabolic adjustments demonstrate that buoyancy regulation in diatoms is an active, energy-dependent process rather than a passive physical response [25].

This rapid and reversible buoyancy control is particularly pronounced in large, non-motile diatoms, which rely on density regulation to adjust vertical position. In contrast, many dinoflagellates primarily depend on flagellar motility for vertical migration, a strategy less directly coupled to short-term nutrient pulses [58], while cyanobacteria often rely on the synthesis or collapse of gas vesicles, a process that typically operates on longer timescales [59]. As a result, selective ion uptake provides diatoms with a fast and energetically efficient mechanism to exploit ephemeral nutrient patches in the surface ocean and modulate carbon export [49,60].

### 3.2. Gas Vesicle Regulation

Phytoplankton can regulate their buoyancy by adjusting the number and size of intracellular gas vesicles, enabling them to remain suspended in the water column or to perform vertical migrations [61,62,63]. This mechanism is particularly important in cyanobacteria, helping them optimize light and nutrient acquisition under varying environmental conditions [64,65]. Cyanobacteria produce gas vesicles, protein-based nanostructures that provide buoyancy without expending energy. Species such as *Anabaena flosaquae*, *Microcystis aeruginosa*, *Planktothrix* sp., and *Anabaena lemmermanni* contain intracellular gas vesicles that enable them to ascend in the water column and form blooms [66,67,68]. Walsby [59] systematically summarized the role of gas vesicles in regulating phytoplankton sinking, noting that cyanobacteria can lower their cell density from 1.04 g cm^−3^ to 0.98 g cm^−3^ by increasing gas vesicle volume, effectively reversing their buoyancy. Gas vesicles have also been observed between the trichomes of *Trichodesmium* colonies, potentially consisting of oxygen bubbles generated through photosynthesis that allow the colonies to float [69]. The formation and stability of gas vesicles are influenced by various environmental factors. Studies have shown that light intensity, nutrient availability, pH, and quorum sensing can all regulate gas vesicle formation and persistence [70,71]. Gas vesicle regulation is thus a critical adaptive strategy that enables phytoplankton to optimize their vertical positioning in the water column.

### 3.3. Biochemical Ballasting Within Cells

#### 3.3.1. Carbohydrate Accumulation and Cellular Densification

The carbohydrate ballasting effect refers to an energy-efficient strategy by which phytoplankton increase cell density and diminish buoyancy through the synthesis and accumulation of dense polysaccharides, thereby enhancing their sinking rates in the water column [49]. Classical experiments have shown that cyanobacteria (*Microcystis*) exposed to high light levels can elevate their polysaccharide content to 2–3 times the baseline level, resulting in a loss of buoyancy and rapid descent [72]. Similarly, the coccolithophore *Emiliania huxleyi* exhibits a substantial increase in intracellular carbohydrate storage, more than doubling its carbon allocation to sugars, which raises its sinking rate from approximately 1 m d^−1^ to between 2 and 4 m d^−1^ [73]. Recent single-cell density imaging studies revealed that multiple green algal species can rapidly ‘weigh down’ by accumulating starch under nitrogen or phosphorus limitation, with sinking rates increasing by 30–300% within just 24 h [57]. However, in diatoms with siliceous shells or specialized morphologies such as *Chaetoceros wighamii* and *Thalassiosira pseudonana*, changes in carbohydrate content have little to no effect on sinking rates, likely due to structural, morphological, and metabolic differences among phytoplankton taxa [74].

In addition, extracellular carbohydrates can spontaneously form transparent exopolymer particles (TEPs) within hours, which promote cellular aggregation, increase particle size, and substantially enhance vertical export fluxes [22,75]. Thus, carbohydrates serve a dual ecological function: intracellularly, they act as ballasting agents that regulate buoyancy at the single-cell level; extracellularly, they serve as adhesive matrices that facilitate aggregation and promote the downward transport of particulate matter. The dynamic production and degradation of carbohydrates therefore exert a critical, twofold influence on the vertical distribution of phytoplankton and the overall efficiency of the biological carbon pump.

#### 3.3.2. Lipid Accumulation and Density Reduction

Lipid accumulation represents a key physiological strategy by which phytoplankton regulate buoyancy and modulate sinking rates, particularly under nutrient-stressed conditions. Lipid classes such as triacylglycerols, mono- and diacylglycerols, and polar phospholipids possess relatively low densities (approximately 0.90–0.93 g cm^−3^), considerably lower than those of other cellular components, such as proteins (1.3–1.4 g cm^−3^) and siliceous frustules (2.1–2.3 g cm^−3^). As a result, the accumulation of lipids can effectively decrease overall cellular density and thereby influence sinking behavior [76,77]. Experimental evidence indicates that nutrient limitation, especially of nitrogen and phosphorus, can trigger a metabolic shift towards enhanced lipid synthesis. For example, Geider and La Roche [78] reported that under phosphorus-depleted conditions, *Thalassiosira pseudonana* increased its lipid content to over 15% of dry weight, accompanied by a significant enhancement in buoyancy. Likewise, Zhang et al. [73] demonstrated a strong positive correlation between lipid content and sinking velocity in *Phaeodactylum tricornutum* (R = 0.91, *p* < 0.001), suggesting that lipids function not only as energy reserves but also as active modulators of vertical positioning in the water column.

However, the effects of lipid accumulation on sinking rates are heterogeneous and sometimes contentious across taxonomic groups. Reynolds [79] noted that in larger diatom species, lipid accumulation alone is often insufficient to counterbalance the ballasting effect of heavy silica frustules, resulting in limited buoyancy enhancement. Similarly, Gemmell et al. [26] observed that in certain large centric diatoms, despite notable lipid accumulation, sinking behavior was primarily governed by cellular metabolic activity rather than density reduction. Therefore, lipid-mediated regulation of phytoplankton sinking is a species-specific and environmentally responsive process. It plays a crucial role in rapid buoyancy adjustment, adaptation to nutrient fluctuations, and reorganization of vertical distribution. Moreover, it represents a significant source of uncertainty in the parameterization of current ocean carbon cycle models [49,80].

### 3.4. Particle Aggregation and Marine Snow Formation

#### 3.4.1. Formation and Sinking of Phytoplankton-Derived Marine Snow

Phytoplankton cells often stick together or with organic debris to form larger aggregates (marine snow), dramatically increasing their effective size and sinking rates [22,32,75]. A primary mechanism driving this aggregation is the production of transparent exopolymer particles (TEPs) from algal exudates. TEPs are colloidal, acidic polysaccharide gels that readily bind dissolved organic matter, bacteria, and cells into larger sticky clumps [32,81,82]. The formation and settling of TEP-rich aggregates can significantly contribute to vertical carbon flux, facilitating the downward transport of POC out of surface waters [83,84,85]. However, TEPs themselves are typically less dense than seawater, with measured densities ranging from 0.70 to 0.84 g cm^−3^ [86]. Without heavier ballast (e.g., calcium carbonate, biogenic silica, or mineral dust) incorporated, TEP-rich flocs often remain suspended near the surface or even become buoyant, limiting their direct contribution to net carbon export [32,87,88]. Thus, while TEPs greatly enhance aggregate formation, their role in driving sinking depends on interactions with denser particles. In natural settings, the sinking behavior of TEP-laden marine snow involves complex processes beyond those observed in lab experiments.

Another key factor is the stickiness of cells and particles, defined as the probability that a collision leads to adhesion. Highly sticky phytoplankton (notably many diatoms) tend to form aggregates readily, whereas less-adhesive cells (e.g., some flagellates) do not [89,90]. Turbulence and shear in the water can increase collision rates, promoting the formation of larger flocs without necessarily causing them to break apart. As particles coagulate into marine snow, sinking velocities rise substantially. For example, aggregates of the mucus-producing alga *Phaeocystis* have been observed to sink at >200 m d^−1^ [91]. Similarly, communities dominated by tiny picoplankton (e.g., *Prochlorococcus*) can form surprisingly cohesive flocs, resulting in higher-than-expected sinking rates. Overall, aggregate size and composition are critical: larger, stickier aggregates with some ballast content sink fastest, whereas loosely bound, porous aggregates or those lacking dense inclusions sink more slowly. Recent experiments demonstrate that marine snow can effectively scavenge even buoyant particles. Notably, phytoplankton aggregates have been shown to incorporate and concentrate microscopic plastic debris, strongly increasing the sinking of microplastics from only tens of meters per day to hundreds of meters per day [3,92]. These findings indicate that marine snow formation is not only central to carbon export but also represents an efficient sink for microplastics, pulling them out of surface waters [93]. However, the impact on the aggregates themselves can vary: in some cases, adding low-density microplastics can reduce an aggregate’s overall density and slow its sinking, whereas in other cases, incorporation of denser plastic fragments can increase sinking velocity. These interactions are an active research area, highlighting the complex ways in which marine snow formation influences not only biological carbon flux but also the fate of pollutants in the ocean.

#### 3.4.2. Microplastic-Phytoplankton Aggregation

Modern studies also show that phytoplankton aggregates play an important role in the vertical transport of microplastics. Hetero-aggregation between microplastics and algae occurs when plastic particles collide with and stick to algal cells or TEP matrices, forming composite particles. Laboratory experiments demonstrate that buoyant microplastic microbeads can become embedded in sinking marine snow, enabling their export to depth [94,95]. Long et al. [93] observed that diatom-derived aggregates readily scavenged 2 µm polystyrene beads, carrying them to deeper layers and effectively removing them from the surface ocean. As a result, microplastic sinking speeds increased from only a few m d^−1^ (as free particles) to over 100 m d^−1^ once captured in marine snow. Field observations likewise report microplastics inside marine snow and even in deep-sea sediments, consistent with aggregate-facilitated sinking as a major removal pathway [94]. However, the presence of microplastics can also alter aggregate properties. For instance, recent studies found that adding low-density plastics to dense diatom aggregates can increase aggregate porosity and slow their sinking, whereas adding higher-density plastics to lighter aggregates can accelerate sinking [94,96,97]. These contrasting effects suggest that the overall impact of microplastics on aggregate sinking depends on the size, density, and concentration of the plastics, as well as the nature of the algal aggregate. Importantly, marine snow provides a biological sink for microplastics, helping explain why observed surface plastic abundances are lower than expected. At the same time, microplastic-laden aggregates may transport plastics into food webs at depth or to the seafloor, raising ecological concerns. In summary, hetero-aggregation between phytoplankton and microplastics can rapidly remove plastics from the upper ocean, but also influences the physical characteristics and sinking behavior of the aggregates themselves.

### 3.5. Ballast Effects at the Aggregate Level

Beyond biochemical adjustments, the incorporation of dense biominerals or other heavy materials can significantly increase aggregate sinking rates. Diatom cell walls made of biogenic silica (opal) and coccolithophore shells of calcium carbonate (calcite) serve as natural ballast in particles. These minerals are far denser (>2.1 g cm^−3^) than organic matter or seawater, and often constitute more than half the mass of sinking particles leaving the euphotic zone [3]. By providing much of the necessary excess density, silica frustules and CaCO_3_ plates make aggregates heavier and sink faster [36]. Similarly, zooplankton fecal pellets are compact parcels rich in absorbed minerals and organic matter; they typically sink on the order of 10–100 s of m d^−1^, which is several orders of magnitude faster than individual phytoplankton cells. The ballast hypothesis of Armstrong et al. [98] proposed that deep-ocean POC flux is tightly coupled to the flux of ballast minerals. Indeed, sediment trap data reveal that the flux of organic carbon at depths > 1500–2000 m is in direct proportion to the flux of associated ballast (opal, carbonate, dust) [99]. In other words, particles that contain more mineral ballast carry more organic carbon to depth, whereas in the absence of ballast, organic matter is more readily remineralized shallower in the water column. Subsequent studies confirmed that regions with higher CaCO_3_ or biogenic silica flux tend to export POC more efficiently [3]. For example, Klaas and Archer [99] found that calcium carbonate-rich particles can enhance deep POC flux by “protecting” organic matter from degradation, whereas in silica-dominated systems, the ballast effect is present but slightly less efficient. In addition, mineral dust can adhere to phytoplankton aggregates, increasing particle density and promoting sinking, and especially relevant during dust deposition events [100]. It is important to note that the relative contribution of different ballast materials can vary: e.g., in high-latitude oceans, silica ballast from diatoms is abundant, while in subtropical gyres, calcite from coccolithophores may dominate. Overall, the presence of heavy ballast particles (SiO_2_, CaCO_3_, lithogenic dust) within aggregates is a major factor enhancing carbon export to the deep ocean [101]. This ballast-driven export helps explain the observed stability of deep POC flux ratios, typically only ~5–7% of total particle mass is organic carbon by 2000 m depth [102]. The ecological implication is that phytoplankton that produce biominerals (like diatoms and coccolithophores), or food webs that generate pellet packaging, have an outsized role in the efficiency of the biological carbon pump [99,100].

## 4. Factors Influencing Phytoplankton Sinking Rates

### 4.1. Intrinsic Cellular Properties

#### 4.1.1. Cell Size, Shape, and Biovolume

According to Stokes’ law, the sinking velocity of a particle is proportional to the square of its radius, making cell size and morphology among the most critical determinants of phytoplankton sinking rates. Because phytoplankton must remain within the upper ocean layers to access sufficient light for photosynthesis, they have evolved various strategies to modulate their sinking behavior. For example, many diatoms, such as *Chaetoceros* and *Skeletonema* possess spines and chain-forming structures that increase their effective surface area, thereby reducing sinking velocity [98]. Some flagellated diatoms extend into chain-like colonies, enhancing their aggregation potential while forming loose, low-density structures that remain suspended in the water column. Conversely, other spiny diatoms can increase mechanical interlocking, forming denser aggregates that sink significantly faster [99]. Laurenceau-Cornec et al. [35] further emphasized that cell morphology not only determines hydrodynamic drag but also systematically influences aggregate architecture, thereby affecting overall sinking efficiency.

In addition to qualitative morphological features such as spines or chain formation, quantitative traits including biovolume, aspect ratio, and specific surface area (SSA) also strongly influence the sinking behavior of phytoplankto [103]. Cells with higher SSA experience greater viscous drag relative to their volume, which slows their descent under low Reynolds number conditions. Irregular or flattened shapes (e.g., discoid diatoms) typically have higher SSA than spherical cells of equivalent volume, leading to slower sinking rates [104]. Biovolume also determines the balance between gravitational and drag forces acting on a cell, making it a key predictor of sinking velocity [67,105]. These morphological and geometric traits have been increasingly used to parameterize phytoplankton sinking in trait-based models and to interpret observed variability in vertical flux. Roselli et al. [106] found that in transitional waters, smaller cells with a higher SSA had a competitive advantage under conditions of nutrient enrichment, suggesting a link between resource availability and cell size distributions. This aligns with findings by Ryabov et al. [103], who highlighted the significant role of cell shape and biovolume in modulating sinking dynamics across different aquatic environments. Furthermore, Stanca et al. [107] demonstrated how environmental factors such as salinity and nutrient availability influence phytoplankton cell morphology, which in turn affects their sinking behavior. These studies emphasize the intricate interplay between cell size, shape, and biovolume in regulating the vertical dynamics of phytoplankton and their role in biogeochemical cycling.

#### 4.1.2. Aggregate Porosity

Porosity is a related structural factor: it refers to the fraction of an aggregate’s volume that is empty space (fluid-filled voids). Phytoplankton-derived aggregates are typically extremely porous, often >90–95% water by volume [24]. High porosity lowers the average density of an aggregate and thus slows its sinking. Larger marine snow particles tend to be more porous (containing more internal voids), forming looser, fluffier structures [42,108]. Although large aggregates usually have greater absolute mass and could sink faster, their very high porosity can counteract some of the ballasting effect if not enough dense material is present [34]. For instance, Bach et al. [109] observed that diatom-dominated aggregates with >80% porosity had limited sinking velocities despite containing heavy silica frustules. On the other hand, when minerals or other heavy particles fill some of the voids, porosity and drag decrease and sinking speeds increase. In summary, intrinsic physical traits, cell size, shape (e.g., spines, chains), and the structural porosity of resulting aggregates, all interact to determine how efficiently phytoplankton biomass sinks. These morphological attributes provide valuable insights into assessing the effectiveness of the biological carbon pump.

#### 4.1.3. Physiological Status

The physiological state of phytoplankton plays a critical role in determining their sinking behavior. Cells in the exponential growth phase are typically metabolically active, exhibit lower densities, and possess greater buoyancy. Conversely, as phytoplankton cells undergo senescence or programmed cell death, the intracellular accumulation of storage compounds such as lipids and carbohydrates leads to increased cellular density, which in turn promotes faster sinking [16,77,110]. Experimental evidence supports these observations. Steele and Yentsch [111] demonstrated that *Thalassiosira nordenskioeldii* in a senescent state showed a marked reduction in sinking velocity after nutrient replenishment. Similarly, Passow [112] reported that *Chaetoceros* species exhibited sinking rates of less than 1 m d^−1^ during active growth phases, while rates could increase to as much as 50 m d^−1^ during the apoptotic phase. Furthermore, low light conditions inhibit photosynthesis and suppress intracellular biosynthetic activity, which also leads to increased sinking rates [53,113].

### 4.2. External Environmental Conditions

#### 4.2.1. Temperature

Temperature influences sinking not only through changes in seawater properties but also by affecting cellular composition and internal density [16,114]. For example, Thompson et al. [115] reported that low temperatures stimulated diatoms to produce more low-density lipids, decreasing cell density by approximately 0.03 to 0.05 g cm^−3^ and increasing buoyancy. In contrast, phytoplankton exposed to high temperatures often accumulate denser compounds such as polysaccharides, which raise cell density and enhance sinking [116]. Bach et al. [31] conducted controlled settling experiments and demonstrated that a 9 °C increase in temperature could raise average particle sinking rates by around 40%. They also projected that a 2 °C rise in sea surface temperature by the end of the century may increase sinking rates by approximately 6%. More recently, Feng et al. [117] showed that warming accelerates buoyancy regulation and daily vertical migrations in the bloom-forming cyanobacterium *Microcystis*, emphasizing the role of temperature in modulating physiological control of sinking. Similarly, Iversen [60] highlighted that predicting phytoplankton sinking under climate change requires integrating both physical viscosity effects and biological adjustments in cellular density. These findings suggest that while warming generally promotes faster sinking in the short term, it may also shift phytoplankton communities toward smaller cells with slower sinking velocities [118,119], with important consequences for the long-term efficiency of the biological carbon pump.

#### 4.2.2. Nutrient Limitation

Limiting nutrients such as nitrogen (N), phosphorus (P), and silica (Si) exert a dual regulatory effect on phytoplankton sinking rates. Early laboratory studies showed that silicon limitation can increase sinking rates by 1.5–4 times in species such as *Skeletonema costatum*, *Chaetoceros gracile*, and *Ditylum brightwellii*, whereas nitrogen and phosphorus limitation typically lead to a 20–60% decline in sinking rates [16]. In coastal waters of the Netherlands, field observations recorded overall sinking rates of 3–5 m d^−1^ under conditions of low phosphate (PO_4_^3−^ < 0.2 µM) and silicate (Si(OH)_4_ < 2 µM) [114]. More recent culture and in situ experiments further support the finding that phosphorus limitation frequently accelerates phytoplankton sinking. In microcosms from the Yangtze Estuary, phosphorus depletion led to an average 40% increase in community sinking rate within 48 h [114]. For *Thalassiosira weissflogii*, phosphorus limitation resulted in an approximately fourfold increase in sinking rate [120]. The large centric diatom *Coscinodiscus wailesii* displayed elevated sinking rates under all three nutrient-limited conditions (N, P, and Si) [18]. Furthermore, silica deficiency may induce under-silicification of frustules and promote cell aggregation, both of which accelerate sinking [121]. Micronutrients, especially iron, also exert a notable influence on sinking dynamics. Muggli et al. [122] reported a fivefold increase in diatom sinking rates under iron stress in the subarctic Pacific, while iron fertilization experiments in the Southern Ocean demonstrated a contrasting reduction in sinking velocity [123]. Together, these findings suggest that nutrient limitation can rapidly modulate phytoplankton sinking, on the scale of hours to days, by altering cell density, frustule silicification, and aggregate formation. This dynamic regulation has profound implications for the short-term efficiency of carbon export in marine systems.

#### 4.2.3. Light Regime

Light availability significantly influences phytoplankton buoyancy regulation and sinking behavior through its control over photosynthetic activity and energy allocation. Under sufficient light, phytoplankton typically synthesize low-density compounds (e.g., lipids) and maintain ionic regulation, thereby reducing cellular density and slowing sinking rates [59,76]. In contrast, extended darkness or low-light conditions suppress photosynthetic energy production, limit buoyancy control, and can lead to the accumulation of denser storage compounds or formation of resting stages, thereby accelerating sinking [53,124]. Both light deficiency and excess can promote sinking, albeit via distinct mechanisms. Low-light conditions may lead to passive sinking due to energy limitation, while high light can trigger carbohydrate overproduction and intracellular densification, such as in *Microcystis* spp. under photo-acclimatory stress [61]. Furthermore, diel vertical migration (DVM) in motile taxa—such as dinoflagellates (*Pyrocystis noctiluca*)—is often synchronized with the light-dark cycle: cells ascend during the day for photosynthesis and descend at night, mediated by circadian ion regulation [125]. At longer temporal scales, seasonal variation in photoperiod and light intensity can impact community composition and vertical flux. During prolonged low-light conditions, non-motile taxa may sink out of the euphotic zone due to insufficient energy for active buoyancy regulation. Additionally, the interaction between light and other environmental factors, such as nutrient availability, can produce synergistic effects. For example, nutrient-depleted cells under light-limiting conditions may rapidly enter senescence or dormancy, enhancing sinking and carbon export [126]. In summary, light modulates phytoplankton sinking by influencing cellular energy budgets and metabolic status. Accurate modeling of sinking behavior requires integrating light regimes with nutrient conditions and species-specific buoyancy mechanisms.

#### 4.2.4. Stratification and Turbulence

**Figure 1:**
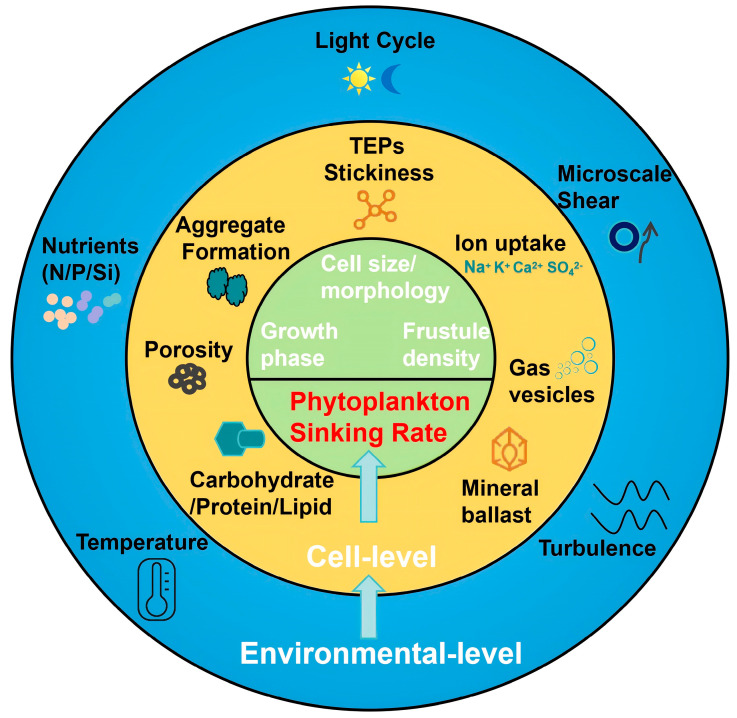
Conceptual diagram illustrating the multiscale mechanisms that regulate phytoplankton sinking rates. The central green core represents intrinsic cellular traits, including size, morphology, and density. The middle orange ring shows cell-derived or cell-mediated processes such as transparent exopolymer particles (TEPs) production, intracellular ballasting, aggregate formation, porosity, and macromolecular composition (carbohydrate, protein, and lipid). The outer blue ring summarizes environmental drivers, including light regime, temperature, turbulence, microscale shear, and nutrient availability. Together, these interacting factors shape phytoplankton sinking behavior and influence the efficiency of carbon export in marine ecosystems.

Stratification and turbulence are dynamic physical processes that modulate phytoplankton vertical distribution and aggregate sinking behavior. In stratified waters, strong density gradients inhibit vertical mixing, reducing nutrient supply from deeper layers and limiting the downward transport of particles. This can prolong phytoplankton residence in the surface layer and suppress carbon export [7,11]. However, reduced turbulence also decreases collision frequencies among particles, thereby limiting aggregation and subsequent sinking. Conversely, moderate turbulence can enhance aggregate formation by increasing particle collision rates, promoting the development of marine snow that sinks rapidly [127,128]. Transparent exopolymer particles (TEPs) and other sticky materials are particularly responsive to shear-induced aggregation under intermediate turbulence levels. However, excessive turbulence can disintegrate fragile aggregates, reducing effective export flux [129,130]. Episodic physical disturbances such as internal waves, storm-driven mixing, or convective overturning can also induce rapid vertical shifts in phytoplankton communities, re-suspend settled particles, or trigger aggregation events. Thermocline depth and seasonal pycnocline development are thus critical in determining both the residence time of cells in the euphotic zone and the vertical transfer of particulate organic carbon [131]. Overall, the interplay between stratification and turbulence governs the size, density, and sinking rate of phytoplankton aggregates, and must be accounted for in models of vertical flux and biological carbon pump efficiency. Figure 1 presents a conceptual diagram illustrating how both cellular and environmental factors regulate phytoplankton sinking rates.

## 5. Methods and Approaches for Measuring Phytoplankton Sinking Rates

Researchers have developed a broad range of methods to quantify phytoplankton sinking rates. These span from classical techniques, such as microscopic cell counting, optical density measurement, and chlorophyll fluorescence, to more recent approaches including the SETCOL method, radioactive tracers, laser scanning, and digital image tracking. Each method has specific advantages and is suited to particular research goals, experimental designs, and technical conditions. For example, Smayda and Boleyn [132] used an inverted microscope to count settling cells, while Steele and Yentsch [111] measured changes in optical density with a spectrophotometer. Eppley et al. [133] and Titman and Kilham [44] assessed biomass loss using chlorophyll a fluorescence. Bienfang [134] introduced a ^14^C tracer technique and later developed the SETCOL method [135], which remains widely adopted in field studies due to its simplicity and low technical demand. More specialized approaches include the salinity gradient technique by O’Brien et al. [136], which tracks vertical motion based on density layers, and the use of laser scanning columns by Walsby and Holland [137]. In recent years, high-resolution tools such as digital microscopy and image-tracking systems have enabled cellular-level analysis of sinking behavior [31,41]. These diverse methods cover multiple spatial and analytical scales, from whole-community assessments to single-cell dynamics. Together, they form a comprehensive framework for studying the mechanisms and variability of phytoplankton sinking. Table 1 provides a summary of each method’s principle, strengths, limitations, and typical applications, offering a practical guide for ecological and biogeochemical research.

Overall, the SETCOL method has been widely applied in field studies due to its operational simplicity, low cost, and suitability for batch measurements under in situ conditions [36,135,141,142]. Although microscopic cell counting is widely used in sinking-related experiments, it is not a direct method for measuring sinking velocity. Instead, it provides basic morphological parameters (e.g., cell size, shape, and abundance) that can be used to infer or model settling rates through theoretical calculations such as Stokes’ law or incorporated into image-based methods. However, recent studies have highlighted that SETCOL tends to systematically underestimate the sinking rates of rapidly settling particles. This underestimation is largely attributed to the use of narrow settling columns (typically <10 cm in diameter), where wall effects can interfere with the descent of large particles or flocculated aggregates, thereby hindering accurate measurements [143]. Additionally, careful timing is required during SETCOL experiments to avoid cell resuspension after settling at the bottom, introducing potential sources of human error. In contrast, in situ imaging techniques or particle image velocimetry (PIV) offer more realistic measurements of sinking velocities under natural conditions, particularly for large aggregates and rapidly sinking particles. These approaches minimize experimental interference and enable real-time monitoring. However, they involve high instrumentation costs and complex data processing, making them less suitable for high-frequency or large-scale field surveys.

In summary, each method carries inherent strengths and limitations. Selection of the appropriate approach should be based on the specific research objectives, particle characteristics, and environmental context. Whenever possible, combining multiple complementary techniques is recommended to overcome the limitations of individual methods and to obtain more reliable and comprehensive estimates of phytoplankton sinking rates.

## 6. Comparative Analysis of Phytoplankton Sinking Rates Between Field and Laboratory Observations

**Figure 2:**
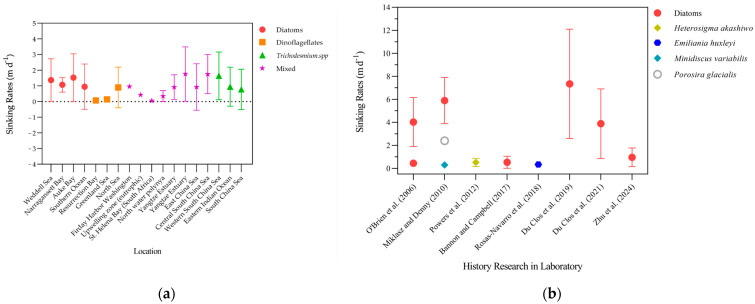
Comparative sinking rates of phytoplankton in field and laboratory studies. (**a**) Sinking rates of natural phytoplankton communities measured in different marine environments, primarily using the SETCOL method. (**b**) Sinking rates of individual phytoplankton species determined under controlled laboratory conditions. Symbols represent the dominant phytoplankton groups, including diatoms, dinoflagellates, cyanobacteria, and mixed assemblages [18,33,38,80,136,153,154,155]. For field data, locations are arranged along major oceanic or estuarine systems, while for laboratory data, species are arranged by taxonomic identity. The dashed line indicates zero sinking rate (neutral buoyancy). Error bars indicate the range of values (minimum–maximum) reported in the original studies, rather than statistical error estimates. Measurement methods and influencing factors are detailed in Supplementary Table S1.

Field-based measurements of phytoplankton sinking rates offer the advantage of capturing particle behavior under natural conditions, thus providing greater ecological relevance for real-world applications. However, they often lack direct access to key particle properties such as density and composition, and typically rely on instruments like underwater cameras and sediment traps. In contrast, laboratory experiments allow precise control of environmental conditions and enable the measurement of a wide range of biological and physical traits. Yet, sampling and handling procedures in the lab may alter the natural heterogeneity of particles [144]. To illustrate the variability across methods and settings, Figure 2 summarizes sinking rates derived from diverse techniques under both field and laboratory conditions. It is important to note that the measurement methods in Figure 2 are not uniform: while field studies predominantly used the SETCOL or trap-based approaches, laboratory estimates often relied on a broader set of tools. Methodological details, including their principles and applicability, are provided in Table 1 and Supplementary Table S1 [145,146,147,148,149,150,151,152,153,154,155]. to enhance comparability across datasets.

Research consistently demonstrates that phytoplankton sinking rates vary widely across spatial scales and community compositions [109,156]. Field studies further show that sinking behavior differs markedly with depth and among oceanic regions [114,152]. In St. Helena Bay, for instance, Pitcher et al. [148] reported that small flagellates dominated under nutrient-poor conditions, whereas diatoms prevailed in nutrient-rich, low-oxygen upwelling zones, leading to elevated sinking rates. Waite and Nodder [123] found that diatom-dominated communities in the Southern Ocean exhibited sinking velocities from −0.50 to 2.40 m d^−1^, with iron acting as a key limiting factor. Alldredge and Gotschalk [24] observed that in situ sinking rates of marine snow were substantially lower than laboratory estimates, likely due to enhanced hydrodynamic resistance associated with high porosity and complex particle morphology. More recently, You et al. [142] applied a modified settling column method during summer surveys in the Yangtze River Estuary and adjacent coastal waters. Reported rates ranged from −0.55 to 2.41 m d^−1^, with higher values recorded outside the river plume front, particularly in phosphorus-limited regions, whereas negative (upward) sinking was more common within the frontal zone near the river mouth.

Different phytoplankton taxa exhibit distinct sinking behaviors. Controlled experiments show that phytoplankton sinking velocities vary by several orders of magnitude among taxa and are strongly influenced by environmental conditions and physiological state. Diatoms generally sink faster than dinoflagellates, primarily due to their denser silica frustules, larger average size, and tendency to form aggregates (e.g., chains or colonies) [19,148]. This is especially true for large diatoms, such as those of the genus *Coscinodiscus*, whose dense silica frustules significantly contribute to higher sinking velocities [38]. However, this general pattern is not universal. Smaller, solitary diatoms may sink more slowly, particularly during nutrient-stressed phases when buoyancy regulation, changes in cellular composition, or carbon accumulation can substantially reduce sinking rates or even lead to near-neutral or upward motion [80]. In contrast, dinoflagellates and cyanobacteria typically exhibit much slower sinking or near-neutral buoyancy, often <1 m d^−1^ or even negative, owing to flagellar motility, gas vesicle regulation, or smaller cell size [19,154]. Imaging-based studies report 0.33–0.57 m d^−1^ for *Skeletonema costatum* [136], about 0.3 m d^−1^ for *Minidiscus variabilis* [38], and 0.16–0.87 m d^−1^ and 0.12–0.55 m d^−1^ for *Heterosigma akashiwo* and *Emiliania huxleyi* [138]. Large centric diatoms of the genus *Coscinodiscus* exhibit the fastest sinking, with values ranging from about 0.86 to more than 12 m d^−1^ depending on species, cell size, and nutrient status [38]. Fluorescence-based tracking of *Thalassiosira pseudonana*, *Coscinodiscus radiatus*, and *Skeletonema marinoi* cultures reported sinking velocities ranging from 0.01 to 1.06 m d^−1^ [80].

These results highlight the importance of size, silicification, and ballast in accelerating descent, and they demonstrate that nutrient depletion, senescence, and light history enhance sinking compared with actively growing cells. Methodological differences also affect reported values, since video tracking and imaging usually yield higher velocities than the bulk SETCOL method, which underestimates sinking due to convection within columns [143].

## 7. Discussion

### 7.1. Ecological and Biogeochemical Implications of Phytoplankton Sinking

Phytoplankton sinking is not only a cellular-level adaptation but also a central mechanism driving oceanic carbon fluxes and nutrient redistribution. The vertical transport of organic matter by sinking cells and aggregates constitutes a major pathway of the biological carbon pump, transferring photosynthetically fixed CO_2_ from the surface ocean to deeper waters or sediments and thereby contributing to long-term carbon storage and climate regulation [60]. In particular, large-scale sinking events toward the end of bloom periods can replenish nutrients in deeper layers while accelerating nutrient depletion in surface waters, with consequences for phytoplankton community succession and zooplankton feeding dynamics.

Differences in sinking behaviour among phytoplankton groups are tightly linked to their functional traits. Distinct taxa (e.g., diatoms, dinoflagellates, cyanobacteria) differ in morphology, density and buoyancy-regulation strategies, leading to large interspecific variation in sinking rates [19]. Fast-sinking diatoms may gain an ecological advantage under high-light or nutrient-depleted conditions by rapidly relocating to deeper habitats, completing life-cycle transitions or escaping competition and grazing. In contrast, motile or buoyancy-regulating species often maintain residence in the euphotic zone, sustaining primary production and favouring recycling within the surface layer. This trade-off between sinking and suspension helps shape vertical distribution patterns, bloom termination pathways and succession dynamics throughout the water column.

The interaction between physical ocean processes and phytoplankton sinking has equally important ecological implications. The persistence of sinking phytoplankton in the photic zone depends on the balance between sinking velocity and turbulent diffusion [30]. When turbulence is weak, cells sink below the euphotic zone and experience light limitation; when turbulence is too strong, vertical structuring is disrupted and residence times in the surface mixed layer become decoupled from intrinsic sinking traits. Maintaining populations of sinking species may therefore require an optimal range of turbulence intensity, which is further modulated by stratification, internal waves and mesoscale circulation. Understanding how these physical–biological interactions vary across regions and seasons is critical for predicting both community composition and carbon export pathways.

### 7.2. Methodological Challenges, Research Priorities and Applied Perspectives

Despite substantial advances, several key challenges remain. First, methodological bias is a major concern. Traditional techniques such as the SETCOL method can underestimate the sinking rates of fast-settling particles and are sensitive to wall effects, secondary circulation and convection, limiting comparability across studies [143]. Refining measurement approaches and validating them with multiple independent techniques, including high-speed imaging, particle tracking, laser-based systems and in situ profilers, is therefore essential. In addition, sediment traps and the ^234^Th disequilibrium method represent the most widely applied techniques for quantifying particle sinking fluxes in natural settings [139,140]. Sediment traps directly collect sinking particles at fixed depths and are suitable for long-term deployments, but they may underestimate fluxes of small, slow-sinking particles due to hydrodynamic biases. The ^234^Th method estimates vertical flux by measuring disequilibrium between ^238^U and its daughter isotope ^234^Th in the water column, offering high sensitivity and large spatial coverage. However, it relies on steady-state assumptions and requires accurate estimates of carbon-to-thorium ratios for flux calibration. Integrating these field-based methods with laboratory approaches and mechanistic models can enhance the accuracy and ecological relevance of sinking-rate estimates. Second, there is a persistent disconnect between laboratory and field observations. Laboratory measurements rarely reproduce the full complexity of natural conditions, where aggregation, grazing, turbulence and chemical micro-heterogeneity vary across scales. Third, the mechanistic basis of sinking remains incompletely resolved: interspecific differences in morphology, cellular composition and physiological state are still not well quantified, hindering the development of trait-based predictive models. Fourth, short-term dynamics are often overlooked. Most studies emphasize quasi-steady conditions, whereas transient environmental changes, such as nutrient pulses, mixing events or light fluctuations, can trigger rapid shifts in sinking behaviour on timescales of hours to days [18]. Capturing these fast responses will require higher-frequency observations and experimental designs that explicitly incorporate variability.

Future research should therefore adopt integrative strategies that link underlying mechanisms to ecological functions and applied outcomes. One priority is to investigate the formation, structure and carbon-sequestration potential of phytoplankton-derived aggregates. Because sinking is strongly shaped by aggregate properties, clarifying the mechanisms and stability of aggregate formation, and how these processes respond to community composition, extracellular polymeric substances and environmental conditions, will improve our understanding of phytoplankton contributions to ocean carbon sinks. A second priority is to resolve species- and trait-specific sinking mechanisms under variable environmental conditions. Systematic experiments exploring how temperature, nutrient regimes, light availability and multiple stressors affect the sinking behaviour of key taxa will clarify how shifts in community composition modulate biological-pump efficiency and will provide valuable constraints for carbon-export models. Third, methodological innovation should continue to enhance measurement accuracy and throughput. Improvements may include increasing SETCOL column diameter to minimize wall effects and convection, using stratified salinity columns to suppress vertical mixing, and coupling laboratory methods with in situ optical and acoustic sensors.

Finally, integrating observations with mechanistic and predictive models that account for interactions among physical, biological and chemical factors is crucial. Such coupled approaches can quantify multi-factorial controls on carbon export and evaluate how phytoplankton sinking will respond to future climate scenarios. Beyond advancing basic plankton ecology, these efforts have important applied implications. A more mechanistic understanding of phytoplankton sinking and aggregation will strengthen ocean carbon-flux models and reduce uncertainty in projections of ocean carbon uptake. At the same time, insights into how phytoplankton functional traits and community structure regulate carbon sequestration can inform nature-based climate solutions, including the design and assessment of artificial carbon-sink interventions and ecosystem-restoration strategies in aquatic environments.

## 8. Conclusions

Phytoplankton sinking constitutes a vital link between cellular-scale biological processes and global biogeochemical cycles. Sinking research spans multiple disciplines, from photosynthetic physiology and cellular biochemistry to marine chemistry and fluid dynamics, and demonstrates that sinking rates are controlled by an interplay of intrinsic traits (e.g., density, morphology, aggregation potential), physiological and ecological context (e.g., nutrient status, life-cycle stage, community interactions) and environmental forcing (e.g., turbulence, shear, stratification). Together, these factors regulate both the efficiency of the biological carbon pump and the structure and succession of phytoplankton communities, with far-reaching ecological and climatic consequences.

Accurate quantification and prediction of phytoplankton sinking therefore require methodological refinement and integrative frameworks that bridge scales. Reducing measurement bias through improved experimental designs and complementary techniques, resolving species-specific mechanisms across variable environmental conditions, and embedding these insights in coupled physical–biogeochemical models will substantially advance the field. By explicitly linking mechanistic understanding of sinking and aggregation to carbon-export and sequestration processes, future research will enhance our capacity to predict the ocean’s role in the global carbon cycle and to evaluate the potential of nature-based strategies for climate mitigation.

## Figures and Tables

**Table 1:** Comparative analysis of methods for measuring phytoplankton sinking rates.

Method Category	Principle	Advantages	Limitations	Applicable Targets	Typical Applications	Reference
Microscopic counting	Periodic cell counts under a microscope to estimate sinking rate	Simple operation; allows morphological identification	Low temporal resolution; labor-intensive	Single species	Static laboratory experiments	[132]
Fluorescence tracing	Estimates sinking speed based on decay of Chl-*a* or probe fluorescence	High-throughput; high sensitivity; compatible with multiwell plates	Cannot distinguish species; depends on algal fluorescence	Cultured populations or communities	High-throughput lab screening	[133]
Radioactive labeling	Tracks ^14^C-labeled cells; infers sinking rate from depthwise radioactivity changes	High sensitivity; suitable for natural communities and dilute samples	Safety concerns; complex operation; restricted usage	Natural communities	In situ field experiments	[134]
SETCOL method	Measures biomass in bottom/layered samples after settling in a transparent column	Easy to use; low cost; works for both monocultures and communities; suitable for biomass quantification	May underestimate rates; sensitive to convection/disturbance; limited resolution	Single species and natural communities	Field and laboratory applications	[135]
Imaging-based tracking	Uses microscopic video to track individual cell motion	High resolution; reveals aggregation and behavioral patterns	Expensive equipment; complex data analysis; unsuitable for bulk measurement	Single cells, chains	Lab-based mechanistic studies	[38]
Laser scanning density column	Tracks vertical cell movement within a stable salinity/density gradient using laser or optics	High precision; avoids disturbance; allows repeated scans	Technically demanding; high cost; complex setup	Density responses of single species	Laboratory physical-ecological studies	[137]
Salinity gradient tracking	Tracks sinking trajectories in a salinity-stratified chamber via particle tracking or manual observation	Simple and visual; compatible with microscopy; good for morphology-based tracking	Not high-throughput; affected by salinity stratification stability	Single species	Lab observation of sinking behavior	[136]
FlowCAM-based imaging	Automatically records particle size, number, and sinking speed via image recognition	High automation; allows continuous tracking of individuals; supports morphological analysis	Image overlap may interfere with accuracy; less effective for low-density samples	Single species or sparse communities	Lab-based fluid column experiments	[138]
Sediment traps	Collect sinking particles in traps moored at fixed depths	Direct field measurement; widely used	Hydrodynamic bias; undersampling of small/light particles	Natural communities; all particle sizes	Vertical particle flux estimation in the field	[139]
^234^Th disequilibrium	Uses ^234^Th/^238^U activity disequilibrium to estimate export flux	Suitable for large-scale flux estimation	Assumes steady-state; requires calibration	Sinking particulate organic matter	Field-based vertical carbon flux estimation	[140]

## Data Availability

No new data were generated in the preparation of this review article. All data discussed are derived from previously published studies, which are cited within the manuscript. Therefore, no dataset is associated with this publication.

## Supplementary Materials

The following supporting information can be downloaded at https://www.mdpi.com/article/10.3390/biology15020130/s1. Table S1: Summary of historical studies on phytoplankton sinking rates in field and laboratory settings.
