# Supplementary material for: A Review of Phytoplankton Sinking Rates: Mechanisms, Methodologies, and Biogeochemical Implications
Source: Biology (Basel). 2026 Jan 12;15(2):130. doi: 10.3390/biology15020130 (PMC12837670; doi:10.3390/biology15020130)
Supplement: Supplementary file 1 [file biology-15-00130-s001.zip › Supplementary Table S1.pdf]

Table S1. Summary of historical studies on phytoplankton sinking rates in field and laboratory settings.

Location	Sinking rate ($m \cdot d^{-1}$)	Dominant phytoplankton	Key influencing factors	Method	Reference
Friday Harbor Washington	0.96	Large and chain-forming diatoms	Nutrients	SETCOL	[145]
Resurrection Bay	0.07	Dinoflagellates	Nutrients	SETCOL	[145]
Upwelling zone (eutrophic)	0.43	Large centric and pennate diatoms	Nutrients	SETCOL	[145]
Weddell Sea	0–2.73	Diatoms	Iron, light	SETCOL	[113]
Narragansett Bay	0.60–1.53	Diatoms	--	SETCOL	[146]
Greenland Sea	0.14	Dinoflagellates	Nutrients, light	SETCOL	[147]
St. Helena Bay (South Africa)	0–0.09	Dinoflagellates and diatoms	--	SETCOL	[148]
Auke Bay, Alaska	0–3.05	Chain diatoms	Nutrients	SETCOL	[149]
Southern Ocean	−0.5–2.4	Diatoms	Iron	SETCOL	[123]
North Sea	−0.4–2.2	Chain diatoms and *Phaeocystis Globosa*	Cell density	SETCOL	[120]
North water polynya	0–0.7	Phytoplankton >5 μm	Nutrients, light	SETCOL	[150]
Yangtze Estuary	0.13–1.71	Diatoms and dinoflagellates	Phytoplankton community composition	SETCOL	[151]
Yangtze Estuary	0.02–3.49	Diatoms and dinoflagellates	Phytoplankton community composition	SETCOL	[141]
Western South China Sea	0.12–3.17	*Trichodesmium* spp.	Phytoplankton community composition	SETCOL	[19]
Eastern Indian Ocean	−0.29–2.19	*Trichodesmium* spp.	Temperature, nutrients	SETCOL	[114]
South China Sea	−0.51–2.07	*Trichodesmium* spp.	--	SETCOL	[36]

East China Sea	−0.55–2.41	Microplankton	Nutrient	SETCOL	[142]
Central South China Sea	0.50–3	Diatoms and cyanobacteria	Cell morphology and size	SETCOL	[152]
Laboratory	0.33–0.57	*Skeletonema costatum*	Cell size	SETCOL, Imaging	[136]
Laboratory	1.9–6.16	*Coscinodiscus* spp.	Cell size	SETCOL, Imaging	[136]
Laboratory	3.9–7.9	*Coscinodiscus spp.*	Cell size	Imaging	[38]
Laboratory	2.4	*Porosira glacialis*	Cell size	Imaging	[38]
Laboratory	0.3	*Minidiscus variabilis*	Cell size	Imaging	[38]
Laboratory	0.16–0.87	*Heterosigma akashiwo*	Growth phase	SETCOL	[153]
Laboratory	0.01–1.06	*Thalassiosira pseudonana, Coscinodiscus radiatus, Skeletonema marinoi*	Cell size	Fluorescence tracing	[80]
Laboratory	0.12–0.55	*Emiliania huxleyi*	Temperature	The Stokes' law	[154]
Laboratory	2.59–12.10	*Coscinodiscus wailesii*	Nutrients	Imaging	[155]
Laboratory	0.86–6.91	*Coscinodiscus wailesii*	Nutrients	Imaging	[18]
Laboratory	0.15–1.77	*Thalassiosira weissflogii*	Nutrients	Imaging	[33]
